# Should Physical Activity Recommendations for South Asian Adults Be Ethnicity-Specific? Evidence from a Cross-Sectional Study of South Asian and White European Men and Women

**DOI:** 10.1371/journal.pone.0160024

**Published:** 2016-08-16

**Authors:** Stamatina Iliodromiti, Nazim Ghouri, Carlos A. Celis-Morales, Naveed Sattar, Mary Ann Lumsden, Jason M. R. Gill

**Affiliations:** 1 Department of Obstetrics and Gynaecology, School of Medicine, University of Glasgow, Glasgow, United Kingdom; 2 Institute of Cardiovascular and Medical Sciences, College of Medical, Veterinary and Life Sciences, University of Glasgow, Glasgow, United Kingdom; Weill Cornell Medical College in Qatar, QATAR

## Abstract

International public health guidelines recommend that adults undertake at least 150 min.week^−1^ of moderate-intensity physical activity. However, the underpinning evidence has largely been obtained from studies of populations of white European descent. It is unclear whether these recommendations are appropriate for other ethnic groups, particularly South Asians, who have greater cardio-metabolic risk than white Europeans. The objective of our study was to determine the level of moderate-intensity physical activity required in South Asians adults to confer a similar cardio-metabolic risk profile to that observed in Europeans of similar age and body mass index (BMI) undertaking the currently recommended levels of 150 min.week^−1^. 148 South Asians and 163 white Europeans aged 18 to 70 years were recruited. Physical activity was measured objectively via vertical axis accelerations from hip-worn accelerometers. Factor analysis was used to summarize the measured risk biomarkers into a single underlying latent “factor” describing overall cardio-metabolic risk. Sex did not modify the association between physical activity and the cardio-metabolic risk factor, so data for both sexes were combined and models adjusted for age, sex, BMI and accelerometer wear time. We estimated that South Asian adults needed to undertake 232 (95% Confidence interval: 200 to 268) min.week^−1^ in order to obtain the same cardio-metabolic risk factor score as a white European undertaking 150 minutes of moderate-equivalent physical activity per week. The present findings suggest that South Asian men and women need to undertake ~230 minutes of moderate intensity physical activity per week. This equates to South Asians undertaking an extra 10–15 minutes of moderate intensity physical activity per day on top of existing recommendations.

## Introduction

There is overwhelming evidence suggesting that migrant South Asians living in high income countries develop type 2 diabetes (T2D) and cardiovascular disease (CVD) almost five to 10 years younger and at lower levels of adiposity than people of white European descent [1–4]. Because of this, lower BMI thresholds to characterize unhealthy body weight in South Asians have recently been adopted by a number of bodies including the American Diabetes Association (ADA) [5] and UK National Institute for Health and Care Excellence (NICE) [6]. Thus it is now well-established that the conventional threshold to define obesity of BMI 30 kg.m^-2^ is not appropriate for all ethnic groups. Like obesity, a low level of physical activity is an important risk factor for T2D and CVD [7–10] and based on a large body of epidemiological and experimental evidence, physical activity guidelines around the world generally recommend that adults undertake at least 150 min.week^-1^ of moderate physical activity (or 75 min.week^-1^ of vigorous physical activity) [11–13]. However these data are largely from studies of white European descent, which has been highlighted as a key limitation in systematic reviews of the literature [7] and physical activity guideline statements [12]. There is evidence that the dose-response relationship between physical activity and health outcomes may not be the same across ethnic groups and that currently recommended levels of physical activity may not necessarily be appropriate for all ethnic groups [14]. In particular, there is accumulating evidence that South Asians may have a ‘low fitness’ phenotype which contributes to their elevated cardio-metabolic risk, and thus may particularly benefit from undertaking higher levels of physical activity [14–16]. Indeed, a recent consensus statement on Physical Activity Guidelines for Asian Indians recommended 30 minutes of moderate-intensity physical activity, 15 minutes of work related physical activity and 15 minutes of muscle strengthening exercises per day [17], but the evidence base underpinning this specific recommendation is unclear. We recently used the approach adopted for the calculation of ethnicity-specific obesity cut-points [18,19] to approximate the level of physical activity needed in South Asian men to confer a similar cardio-metabolic disease risk profile as white European men undertaking 150 minutes of moderate physical activity per week [20] [21]. However, it is not known whether these findings extend to South Asian women, who have particularly high T2D and CVD risk [22–25] and are typically highly inactive [26,27].

The aim of this study was therefore to determine the level of physical activity required in South Asian women and men living in the UK to confer an equivalent cardio-metabolic disease risk profile to white Europeans undertaking the current guideline level of physical activity.

## Material and Methods

### Participants

Data on South Asian (defined as having both parents of Indian, Pakistani, Bangladeshi or Sri Lankan origin) and European women (both parents of white European descent) were collected from a cross-sectional study conducted in Scotland. Participants were recruited mainly through general advertising and word of mouth. Participants were not compensated for their time. Women aged between 18 to 70 years, lived in the UK and did not have a history of CVD, diabetes or polycystic ovary syndrome. Women on hormone replacement therapy or hormonal contraceptives were excluded. Only women with valid data for objectively monitored physical activity were included in this analysis. Data were combined with valid data from men that were recruited for the Carotid Ultrasound and Risk of Vascular disease in Europeans and South Asians (CURVES) study which has been described extensively elsewhere [20]. Participants’ health history, including smoking status was determined by questionnaire.

### Ethics, consent and permissions

Both studies were approved by the West of Scotland Research Ethics Committee and conducted according to the Declaration of Helsinki. All participants gave written informed consent to participate.

### Physical Activity

Participants wore accelerometers (Actigraph G3TX+ or Actitrainer, ActiGraph LLC, FL, USA) at all times except when showering, swimming and sleeping for seven consecutive days. Vertical axis accelerometer counts were summarised into 60-second epochs and activity intensity domains were classified based on the Freedson cut-points [28]. Valid data were considered when participants wore the accelerometers for at least 4 days for a minimum of 10 hours each day. Non-wear time was defined as the intervals of at least 60 min of zero activity. Weekly moderate-equivalent physical activity was defined as the summation of the time spent performing moderate activity plus the time spent performing vigorous activity multiplied by two (based on the Freedson criteria), in line with the 2:1 weighting of vigorous vs moderate physical activity in current guidelines (i.e. 75 min vigorous physical activity is considered equivalent to 150 min moderate physical activity per week) [11–13]. Moderate-equivalent activity was used as the activity variable in the data analysis to estimate ethnic-specific cut points. In addition, current guidelines state that physical activity should be performed in bouts of at least 10 min duration [11–13]. Therefore, physical activity of at least moderate intensity (i.e. moderate-to-vigorous activity, MVPA) undertaken in bouts of 10 min duration with an allowance within a bout for interruption up to 2 min below the moderate intensity threshold [29] was also calculated (MVPA_bouts_). This was the second activity variable (exposure) for additional analysis.

### Biochemical markers

Venous blood samples were collected after an overnight fast. Glucose and glycated haemoglobin (HbA1c) were analysed as routine samples on the day of collection in one of the certified NHS Biochemistry laboratories within Greater Glasgow and Clyde by using standard automated enzymatic and HPLC techniques. Centrifuged serum and plasma were stored at -80°C for subsequent analysis. Lipids (total cholesterol, high density lipoprotein (HDL-c), triglycerides (TG) were measured in thawed sera using automated enzymatic technique at the end of the study. LDL levels were calculated with the use of the Friedewald equation [30]. Insulin was measured in stored plasma by using a commercially available ELISA (Mercodia AB, Uppsala, Sweden) after the completion of the study.

### Statistical Analysis

Statistical analysis was performed using STATA package (version 12.1, StataCorp LP, USA). Using a similar approach described previously [18–20], factor analysis was used to summarise cardio-metabolic risk variables into a single variable. Glycaemia variables (HbA1c, fasting plasma glucose), lipid/insulin resistance variables (HDL-c, triglycerides, total cholesterol and insulin) and blood pressure (systolic and diastolic blood pressure) were included in the factor analysis. These variables were chosen as established biomarkers with strong associations to incident cardiovascular and metabolic disease [31]. Biomarkers with rotated loadings >0.32 for principal components (i.e. explaining >10% of the variance in a factor (0.32^2^  =  0.10)) in these analyses were clustered into summary factors. The single summary factor that accounted for the largest proportion of variation of the variables was selected as the dependent variable. This included the standardized levels of HbA1c, glucose, HDL-c, TG, insulin, total cholesterol, systolic and diastolic blood pressure. Regression models were fitted with the summary factor as dependent variable and either total moderate-equivalent physical activity or MVPA_bouts_ as the exposure variables. In addition, all models contained the interaction of ethnicity with the independent variable or the interaction of sex with the independent variable. If the interaction terms did not improve the fitting of the model, then the interaction term was not included in the model and sex was included as a covariate. In addition, the models were adjusted for age, accelerometer wear time, BMI and smoking status. Statistical significance was adopted at p < 0.05.

## Results

311 out of 364 participants (85.4%) (n = 153 of 178 women (86.0%) and n = 158 of 186 men (85.0%)) had valid accelerometer data. There were no marked differences in baseline characteristics and key variables between the participants with valid and without valid accelerometer data (data not shown). Table 1 shows detailed descriptive data in both women and men directly relevant to the present analyses. In summary, both groups had similar age and BMI. S1 Table shows descriptive characteristics of women, whereas data in men have been presented elsewhere [20]. South Asians were less active and showed a more insulin resistant phenotype with greater levels of fasting insulin, HbA1c and TG and lower levels of HDL-c compared with the Europeans.

**Table 1:** Descriptive characteristics of the cohort.

Characteristics	South Asians N = 148	Europeans N = 163	p-value
**Women (n, %)**	73 (49.3)	80 (49.1)	
**Age (years)**	49.0 (42.0, 55.0)	49.0 (44.0, 55.0)	0.58
**Body mass (kg)**	75.1 ± 14.4	77.0 ± 16.1	0.59
**Height (m)**	1.66 ± 0.1	1.71 ± 0.1	0.0002
**BMI (kg.m**^**-2**^**)**	27.1 ± 4.6	26.3 ± 4.4	0.11
**Waist circumference (cm)**	88.2 (80.0, 101.0)	86.5 (76.3, 95.6)	0.05
**Current smokers n (%)**	8 (5.4)	8 (4.9)	0.84
**Moderate-to-vigorous physical activity (min.week**^**-1**^**)**	166.3 (79.9, 297.2)	317.0 (197.4, 495.0)	0.0001
**Moderate to vigorous physical activity measured in bouts (min.week**^**-1**^**)**	20.0 (0, 92.0)	40.0 (111.0, 235.0)	0.0001
**Accelerometer wear time (hours.day**^**-1**^**)**	13.9 (12.8, 14.8)	14.4 (13.7, 15.1)	0.0006
**Glucose (mmol.l**^**-1**^**)**	5.0 (4.7, 5.6)	4.9 (4.6, 5.3)	0.04
**HbA1c (mmol.mol**^**-1**^**)**	38.0 (35.0, 41.0)	34.0 (32.0, 37.0)	0.0001
**HbA1c (%)**	5.6 (5.4, 5.9)	5.3 (5.1, 5.5)	0.0001
**Insulin (mmol.l**^**-1**^**)**	11.2 (7.2, 15.6)	7.6 (5.1, 10.1)	0.0001
**Total Cholesterol (mmol.l**^**-1**^**)**	5.2 ± 0.9	5.5 ± 0.9	0.03
**HDL cholesterol (mmol.l**^**-1**^**)**	1.3 (1.1, 1.5)	1.5 (1.2, 1.8)	0.0001
**Triglycerides (mmol.l**^**-1**^**)**	1.2 (0.8, 1.8)	1.0 (0.7, 1.4)	0.02
**Systolic Blood Pressure (mmHg)**	124.0 (116.0, 134.0)	124.0 (115.0, 134.0)	0.80
**Diastolic Blood Pressure (mmHg)**	77.0 (71.0, 85.0)	76.0 (71.0, 83.0)	0.43
**Overall Cardio-metabolic Factor (SD)**	0.45 (-0.48, 0.96)	-0.30 (-0.84, 0.36)	

Data are presented as mean ± standard deviation or median (interquartile range)

### Total moderate-equivalent activity versus MVPA_bouts_

Ethnicity and sex did not modify the associations between activity variables and the summary cardio-metabolic risk factor; hence data from all participants were included in the same model. Table 2 shows the change in the cardio-metabolic risk factor with one standard deviation increase in the activity variables. In univariate models there were significant associations between both total moderate-equivalent activity and MVPA_bouts_ with the cardio-metabolic risk factor. Both associations were attenuated by adjustment for age, BMI, ethnicity, sex, smoking and total accelerometer wear time. However, while the association of total moderate-equivalent physical activity and the cardio-metabolic risk factor remained significant after adjusting for confounders, the association of MVPA_bouts_ and the cardio-metabolic risk factor was substantially attenuated by adjustment and lost statistical significance. In the unadjusted models, total moderate-equivalent activity explained 18.4%, whereas MVPA_bouts_ explained 12.6% of the variance in the summary cardio-metabolic risk factor. Therefore, total moderate-equivalent activity (rather than MVPA_bouts_) was used as the exposure in estimating the equivalent levels of physical activity to 150 min.week^-1^ for the South Asians. Overall, the fully adjusted model including total moderate-equivalent activity, age, BMI, ethnicity, sex, smoking and total accelerometer wear time variance in the cardio-metabolic risk factor explained 44% of the variance in the cardio-metabolic risk factor. BMI and smoking, the other modifiable risk factors included in the fully adjusted model, explained 8% and 2.4%, respectively, of the variance the cardio-metabolic risk factor in univariate models.

**Table 2:** Change in the summary cardio-metabolic factor for one standard deviation increase in weekly moderate-equivalent physical activity or in moderate-to-vigorous physical activity undertaken in bouts of ≥ 10 min. The adjusted model is adjusted for age, BMI, ethnicity, sex,total accelerometer wear time, smoking.

	Standardized b coefficient (95% CI)(univariate model)	Standardized b coefficient (95% CI)(adjusted model)	p-value(univariate model)	p-value(adjusted model)	p-value interaction with ethnicity	p-value interaction with sex
**Moderate-equivalent physical activity (min.week**^**-1**^**)**	-0.43(-0.53 to -0.33)	-0.20 (-0.30 to -0.10)	<0.001	<0.001	0.09	0.63
**Moderate-to-vigorous physical activity in bouts of ≥10 min (min.week**^**-1**^**)**	-0.35 (-0.45 to -0.25)	-0.09 (-0.19 to 0.01)	<0.001	0.08	0.14	0.18

### Equivalent levels of physical activity

**Fig 1:**
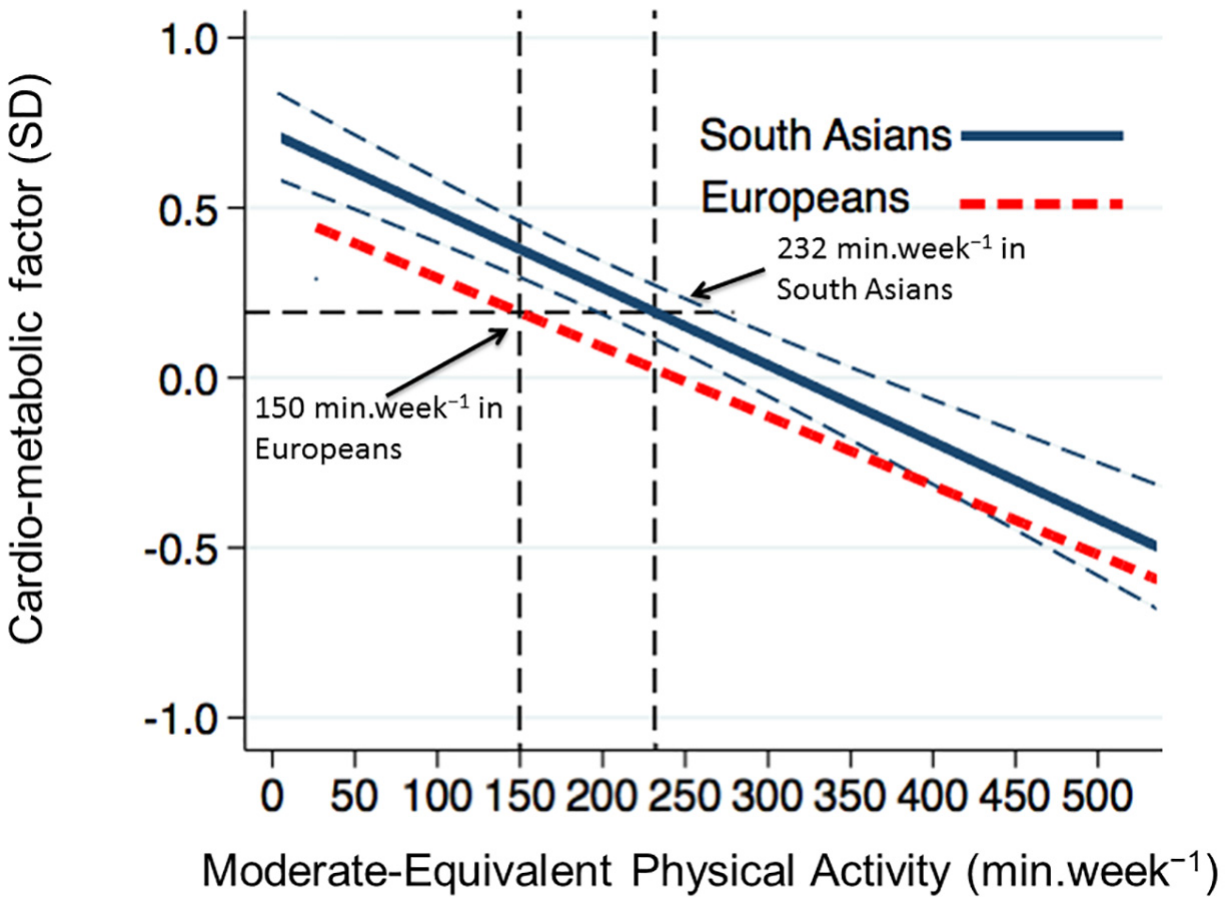
Relationship between the overall cardio-metabolic risk factor and level of moderate equivalent physical activity in South Asians (solid blue line) and Europeans (solid red line). 232 (200 to 268) min.week^−1^ of moderate equivalent physical activity in South Asians gave an equivalent cardio-metabolic risk factor level to that observed in Europeans undertaking 150 min.week^−1^. Thinner blue lines represent the 95% confidence bands around the regression line for South Asians. These bands were used to calculate the 95% CI around the equivalent level of physical activity in South Asians. Regression lines are adjusted for age, BMI, total accelerometer wear time, sex and smoking.

The analyses suggest that South Asian adults need to undertake 232 min.week^-1^ (95% CI: 200 to 268) of moderate-equivalent physical activity to achieve the same cardio-metabolic risk factor value as European adults undertaking 150 min.week^-1^ moderate-equivalent physical activity, in analyses adjusted for age, BMI and accelerometer wear time. There were no significant physical activity x sex, or physical activity x ethnicity x sex interactions with the cardio-metabolic risk factor outcome, indicating that the required level of physical activity in South Asians for equivalent risk to Europeans did not differ between men and women. Fig 1 illustrates how these threshold values were calculated.

## Discussion

This study demonstrates that South Asians of both sexes need to undertake higher levels of physical activity to exhibit an equivalent cardio-metabolic risk profile to that of adults of white European descent of similar age and BMI achieving current physical activity guidelines (232 min.week^-1^ versus 150 min.week^-1^ of moderate equivalent physical activity respectively). These data from a mixed-sex cohort extend our previous observations in men [20], building the case for evidence-based ethnicity-specific physical activity guidelines and importantly show that similar levels of physical activity could be recommended for South Asian women and South Asian men. These data are particularly important since, at present, South Asians engage in less activity than do white Europeans.

A clear observation was that total moderate-equivalent physical activity, including activity bouts of all durations, had a stronger association with cardio-metabolic risk profile than MVPA performed in bouts of at least 10 minutes. This suggests that current recommendations to undertake physical activity in bouts of at least 10 minutes may not be necessary or appropriate and is in line with other studies which have shown that physical activity accumulated in very short bouts is associated with lower cardio-metabolic disease profile [32], CVD and all-cause mortality [9,33]. Interestingly, overall levels of physical activity in our cohort differed markedly depending on whether all physical activity or physical activity in 10-minute bouts was considered. Only 15% of South Asians and 41% of Europeans in our cohort achieved at least 150 minutes of MVPA per week measured in 10 min bouts, whereas 55% of South Asians and 84% of Europeans achieved this target when all moderate-equivalent physical activity was included. Based on other data using accelerometer-based physical activity assessments in white European adults, the physical activity levels in our study cohort appear to be broadly equivalent to other studies in high income countries [29,34]. Interestingly, while physical activity levels were generally lower in South Asians than Europeans in our sample, in line with other observations [35], 33% of South Asians achieved the target of 232 min.week^-1^ of moderate-equivalent physical activity, suggesting while achieving this target is likely to be a challenge for many South Asians, a sizable minority were already reaching this threshold.

The concept of ethnicity specific recommendation for physical activity has been discussed elsewhere. A recent study in Sri Lankan women demonstrated women who self-reported less than 2,640 MET-minutes.week^-1^ of moderate to vigorous physical activity (~400–800 minutes per week of moderate intensity physical activity) are more likely to exhibit impaired glycaemia compared to women with self-reported weekly activity greater than this threshold (sensitivity 84% and specificity 85%) [36]. Although that study supports the concept of greater physical activity requirements for South Asian women, it is important to recognize that the use of self-reported questionnaires substantially overestimates physical activity [37] and limits the analysis exploring the actual dose-response relationship between physical activity and glycaemia. A consensus report developed in India proposed the idea that South Indians need to perform 30 min of moderate intensity physical activity, 15 min of work related activity and 15 min of weight bearing exercises daily with the summary physical activity exceeding the current recommendations of 150 min.week^-1^ [38], however it is unclear how these recommendations were developed and the evidence behind them. Our previous analysis exclusively in men indicated that South Asian men needed to undertake 266 (228 to 313) min.week^-1^ of physical activity performed in bouts of 10 min or 384 (231 to 536) min.week^-1^ of total moderate physical activity (unpublished data for moderate physical activity) in order to equate their overall cardio-metabolic risk with that of men of white European descent [20]. The present analysis in both men and women (without sex being a significant effect modifier of the association between physical activity and cardio-metabolic factor) indicate that South Asian adults should undertake at least 232 min.week^-1^. This level of activity is within the confidence intervals of the earlier analysis limited to men, and the larger sample size here enables the physical activity estimate to be made with greater precision than our previous analysis. However, direct comparison with the previous estimated recommended time for South Asian men cannot be made as we use a more adjusted model in our index analysis and our exposure is moderate-equivalent physical activity rather than moderate-to-vigorous physical activity or bouts of at least 10 minutes duration.

### Strengths and weaknesses

A key strength of this study is that physical activity was measured with the use of an objective measure which is essential in quantifying the real dose-response relationship between physical activity and cardio-metabolic risk. In contrast, self-report measures of physical activity have been shown to be imprecise and to substantially overestimate activity levels, which has the consequence of masking the true magnitude of the association between activity and risk [37]. Although accelerometers can underestimate the level of activity associated with load-carrying, cycling or swimming, the participants in this study kept a log of their activities and only a minor proportion of the participants performed any of the above activities; thus it seems unlikely that this would have substantially biased our results. The factor analysis strategy used in this study is an established methodology that aggregates relevant biomarkers to define cardio-metabolic risk, which is relatively independent of the distribution of a variable and takes into consideration the continuous nature of a variable without dichotomizing them [39]. In contrast, alternative methods of receiver-operating characteristic curves (ROC) and logistic regression have shortcomings; both are restricted to study dichotomous outcomes and ROC analysis is subjected to the distribution of a variable in the study population [39].

It is important to recognize that the evidence base underpinning the current 150 min.week^-1^ was largely based on studies in which physical activity was self-reported, rather than objectively measured. Accelerometer-measured physical activity is typically lower than self-reported values—in one estimate by 2.5-fold [37]–thus 150 min.week^-1^ of self-reported physical activity could conceivably be equivalent to ~60 min.week^-1^ of accelerometer-measured physical activity. Re-running our analysis using 60 min.week^-1^ as the guideline amount of physical activity, results in an equivalent estimate for south Asians of ~140 min.week^-1^. Thus, using either approach, south Asians would need to undertake ~80 min/week more (accelerometer measured) physical activity (232 mins vs 150 mins or 140 mins vs 60 mins) than white Europeans for a similar cardio-metabolic risk profile. This corresponds to ~15 minutes additional physical activity per day if individuals are active on 5 days of the week.

The present study has some limitations. It is cross-sectional with a relatively modest sample size assuming that increasing physical activity in South Asians will moderate their background cardio-metabolic risk. Hence, randomised controlled trials (RCTs) examining whether South Asians undertaking the proposed levels of physical activity would exhibit a similar cardio-metabolic profile with that of white Europeans of similar age and BMI exercising for 150 min.week^-1^ are warranted before definitive recommendations regarding ethnicity-tailored physical activity can be implemented.

Nevertheless, the present work makes an important contribution to the evidence-base for ethnicity-specific physical activity recommendations, by extending previous observations to women and by increasing the precision of the estimate for the level of physical activity required in South Asian adults for equivalent risk. We acknowledge that the South Asians in this study may not be representative of all South Asians living in the UK (i.e. we excluded people with diabetes and only included specific age groups). Whilst this would limit the ability to generalize prevalence rates (which is beyond of the scope of this study), estimates of the magnitude of associations between physical activity and CVD risk will not be affected by this and will therefore be generalizable. Thus, the recent recommendation from the Joint British Societies’ consensus recommendations for the prevention of cardiovascular disease that South Asian men may benefit from higher levels of physical activity than currently recommended [21], can reasonably be extended to South Asian women based on the present data.

## Conclusion

The present findings suggest that South Asian men and women need to undertake ~230 minutes of moderate intensity physical activity per week to confer a similar cardio-metabolic risk profile to adults of white European descent achieving current physical activity recommendations of 150 minutes per week. This equates to South Asians undertaking an extra 10–15 minutes of moderate intensity physical activity per day. While an RCT is needed to demonstrate whether these levels of physical activity would moderate the cardio-metabolic risk of South Asians, these data highlight the need of educating South Asians of the importance of physical activity and the development of culturally appropriate strategies to increase physical activity in this ethnic group.

## Supporting Information

S1 Table Descriptive characteristics of the cohort of women. (DOCX)
