# Supplementary material for: Should Physical Activity Recommendations for South Asian Adults Be Ethnicity-Specific? Evidence from a Cross-Sectional Study of South Asian and White European Men and Women
Source: PLoS One. 2016 Aug 16;11(8):e0160024. doi: 10.1371/journal.pone.0160024 (PMC4987009; doi:10.1371/journal.pone.0160024)
Supplement: S1 Table — (DOCX) [file pone.0160024.s001.docx]

**S1 Table**

Descriptive data of the cohort of women

Data are presented as mean ± standard deviation or median (interquartile range)

| **Characteristics** | **South Asian women**  **N= 73** | **European women**  **N=80** | **p-value** |
| --- | --- | --- | --- |
| **Age (years)** | 50.6 (43.2, 57.8) | 50.0 (38.0, 55.8) | 0.86 |
| **Body mass (kg)** | 67.9 ± 13.4 | 67.4 ± 11.6 | 0.93 |
| **Height (m)** | 1.59 ± 0.1 | 1.63 ± 0.1 | 0.0001 |
| **BMI (kg.m^-2^)** | 26.4 (22.8, 29.5) | 24.9 (22.3, 27.5) | 0.1 |
| **Waist circumference (cm)** | 80.5 (73.0, 88.3) | 76.3 (70.8, 81.4) | 0.005 |
| **Moderate-to-vigorous physical activity (min.week^-1^)** | 172.0 (64.0, 318.0) | 381.6 (219.0, 509.5) | 0.001 |
| **Moderate to vigorous physical activity measured in bouts (min.week^-1^)** | 20.0 (0, 144.0) | 160.5 (59.0, 261.5) | 0.001 |
| **Accelerometer wear time (hours.day^-1^)** | 13.9 ± 1.4 | 14.3 ± 1.2 | 0.04 |
| **Number of valid days of accelerometer wear (days)** | 7 (6, 7) | 7 (6, 7) | 0.10 |
| **Glucose (mmol.l^-1^)** | 4.8 (4.5, 5.3) | 4.8 (4.5, 5.1) | 0.56 |
| **HbA1c (mmol.mol^-1^)** | 37.0 (33.0, 41.0) | 33.0 (31.0, 36.0) | 0.001 |
| **HbA1c (%)** | 5.5 (5.2, 5.9) | 5.2 (5.0, 5.4) | 0.001 |
| **Insulin (mmol.l^-1^)** | 9.9 (5.8, 14.5) | 7.2 (4.9, 9.3) | 0.02 |
| **Total Cholesterol (mmol.l^-1^)** | 5.1 ± 0.9 | 5.3 ± 0.8 | 0.17 |
| **HDL cholesterol (mmol.l^-1^)** | 1.4 (1.2, 1.7) | 1.7 (1.5, 2.0) | 0.0001 |
| **Triglycerides (mmol.l^-1^)** | 1.0 (0.8, 1.6) | 0.8 (0.7, 1.2) | 0.02 |
| **Systolic Blood Pressure (mmHg)** | 124.0 (108.0, 136.0) | 120.5 (112, 134) | 0.92 |
| **Diastolic Blood Pressure (mmHg)** | 78.0 (67.0, 85.0) | 78.0 (50.5, 83.0) | 0.64 |
